# Supplementary material for: CMOS-Compatible High-Performance Silicon Nanowire Array Natural Light Electronic Detection System
Source: Micromachines (Basel). 2024 Sep 27;15(10):1201. doi: 10.3390/mi15101201 (PMC11509308; doi:10.3390/mi15101201)
Supplement: Supplementary file 1 [file micromachines-15-01201-s001.zip › micromachines-3159520-supplementary.pdf]

micromachines

Supporting Information

CMOS-Compatible High-Performance Silicon Nanowire Array Natural Light Electronic Detection System

Xin Chen [1,†], Jiaye Zeng [1,†], Mingbin Liu [1], Chilin Zheng [1], Xiaoyuan Wang [2], Chaoran Liu [3] and Xun Yang [1,*]

[1] School of Electronic and Information Engineering, China West Normal University, Nanchong 637002, China; cxing202303@163.com (X.C.); 18783008545@163.com (J.Z.); liumb926@163.com (M.L.); clzheng925@163.com (C.Z.)
[2] Zhejiang Key Laboratory of Ecological and Environmental Big Data, Hangzhou 321001, China; wangxiaoyuan@zjemc.org.cn
[3] Ministry of Education Engineering Research Center of Smart Microsensors and Microsystems, College of Electronics and Information, Hangzhou Dianzi University, Hangzhou 310018, China; liucr@hdu.edu.cn
* Correspondence: yangxun@cwnu.edu.cn
† These authors contributed equally to this work.

The another structures of SiNW natural light detectors:

We use the same method to prepare a new SiNW array natural light detector with smaller area and lower power consumption. There are 12 nanowires in the new array, the detector has an area of only 0.18 mm^2, a smaller area means more production and lower cost. Due to fewer SiNWs connected in parallel between the positive and negative electrodes, the resistance of detector is higher (~10MΩ), the working current is 0.35 μA, the power consumption is reduced to 0.175 μW.

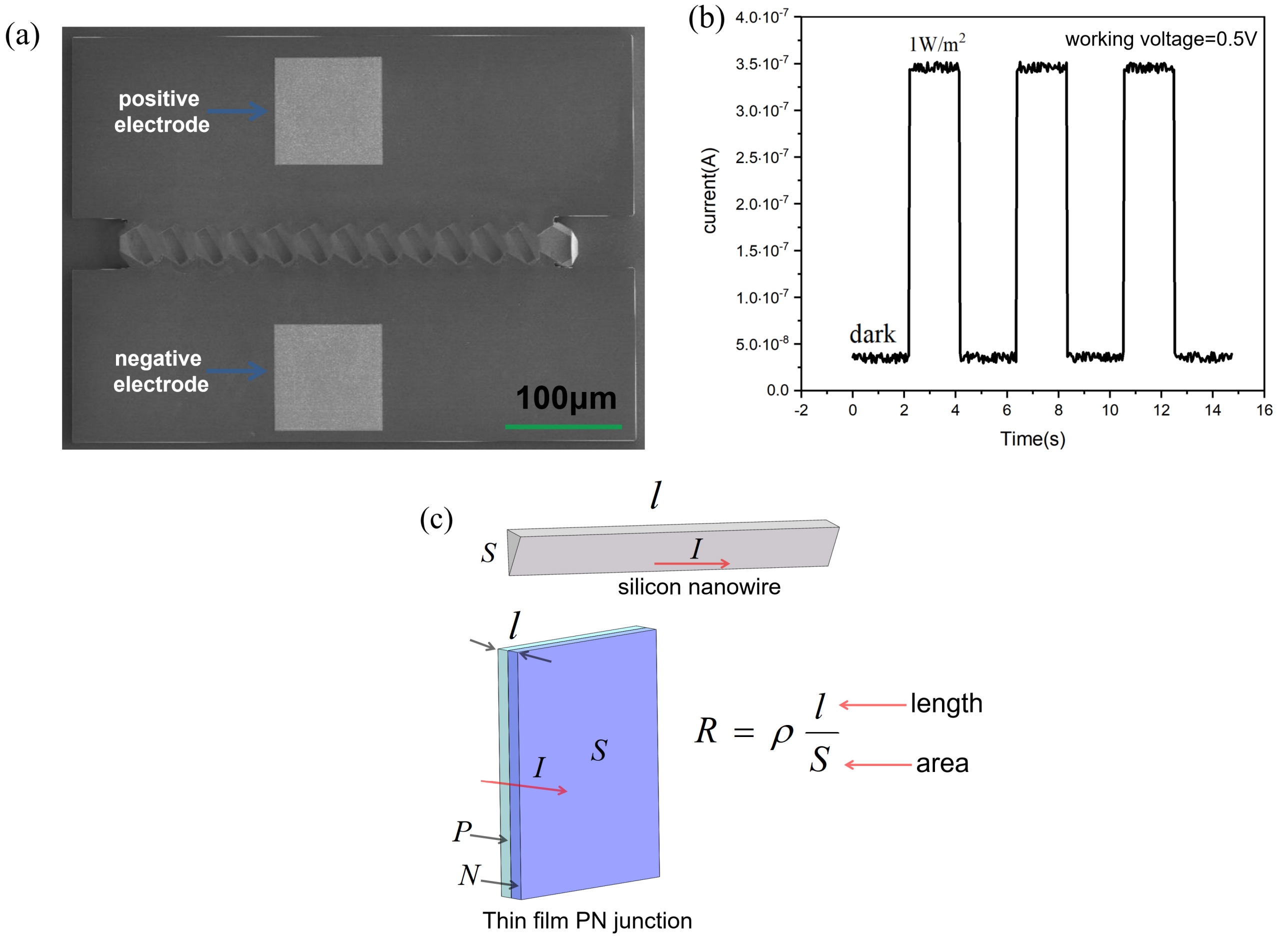

Figure S1. (a) the electron microscopy image of the SiNW array natural light detector; (b) the current signal of the detector in dark environment and 1 $W{\cdot}m^{-2}$ light intensity environment; (c) the models of SiNWs and thin film PN junction.

Figure S1 c shows the reason why the slender morphology of SiNW can bring low power consumption, the resistance of the detector is inversely proportional to the length and proportional to the cross-cutting area, so the resistance of the SiNW is very small, that results in low working current and low power consumption. For planar photodetectors, the situation is completely opposite, in order to perceive light, the area of the planar photodetectors will not be too small.

The calculation of the responsivity R:

$$R = \frac{I_{ph}}{P \cdot S} = \frac{1.093 \cdot 10^{-6}(A)}{0.5(W/m^2) \cdot 5.852 \cdot 10^{-7}(m^2)} \approx 3.74(A/W)$$

I_{ph} represents the net photocurrent, P represents the light intensity, and S represents the effective irradiation area of the detector. The light intensity P is 0.5 W/m^2, the net photocurrent of the device is 1.093 μA, and the irradiation area is 5.852×10^7 m^2.

The relationship between the detectivity D* and the light intensity P:

First of all, we observe that the photocurrent and light intensity are not directly proportional. With the increase of light intensity, the photocurrent tends to be saturated (Figure 4b). In order to clarify the relationship between photocurrent and light intensity, we fitted the measured experimental data using a power law fitting formula[20].

$$I_{ph} \propto P^{\theta}$$

Where I_{ph} represents the net photocurrent, P represents the light intensity, θ represents the empirical value related to the photogenerated carrier composite activity, which can be used to describe the degree of photogenerated carrier composite. As shown in Figure 4(b), according to the fitted result, the value of θ is about 0.7 (not proportional), which is a deviation from the ideal value (θ=1), it indicates the existence of photogenerated carrier recombination in the detector, the phenomenon may be caused by trap states between the Fermi level and the conduction band edge[21,22].

The net photocurrent I_{ph} will affect the responsivity R:

$$R = \frac{I_{ph}}{P \cdot S}$$

Further, the responsivity R will affect the detectivity D*:

$$D^* = R \cdot \frac{S^{1/2}}{(2 \cdot e \cdot I_{dark})^{1/2}}$$

According to the above formula, the physical mechanisms of the relationship between detectivity and light intensity is related to the recombination of photogenerated carriers. To further clarify their quantitative relationship, we introduce θ=0.7 into the first equation：

$$I_{ph} = k \cdot P^{0.7} \quad \text{(k is the scale factor)}$$

$$R = \frac{I_{ph}}{P \cdot S} = \frac{k \cdot P^{0.7}}{P \cdot S} = \frac{k}{P^{0.3} \cdot S}$$

$$\rightarrow D^* = \frac{k}{P^{0.3} \cdot S} \cdot \frac{S^{1/2}}{(2 \cdot e \cdot I_{dark})^{1/2}} = P^{-0.3} \cdot \boxed{\frac{k}{(2 \cdot e \cdot S \cdot I_{dark})^{1/2}}} \longleftarrow \text{all constant=A}$$

$$D^* = A \cdot P^{-0.3}$$

In short, due to the recombination of photogenerated carriers, the relationship between the detectivity D* and the light intensity P is negative exponential power function, which explains why the detectivity D* decreases as the detectivity P increases.

The comparison of signal intensity at different incident angles:

As shown in Figure S2 a, the large angle incident light illuminates the SiNW, and then illuminates the bottom and side wall of the groove, the light will be reflected, so that the SiNW will be illuminated again, that is why the signal of the large angle incident light is stronger. On the contrary, when small angle incident light illuminates detector, only a

small part of the light enters the groove for reflection, the effect of secondary irradiation is not obvious, that is why the signal of the small angle incident light is smaller.

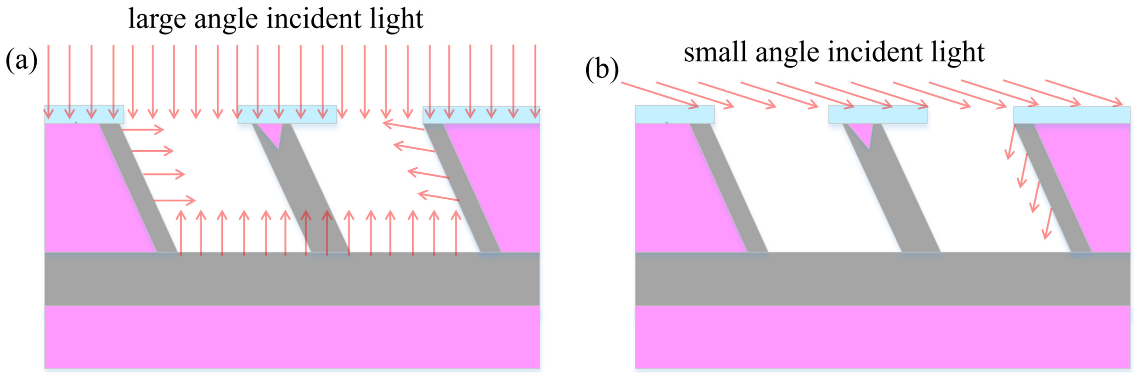

Figure S2. The comparison of different incident angles. (a) the large angle incident light; (b) the small angle incident light.
